# Small RNA-Seq Analysis Reveals miRNA Expression Dynamics Across Tissues in the Malaria Vector, *Anopheles gambiae*

**DOI:** 10.1534/g3.119.400104

**Published:** 2019-03-07

**Authors:** William Bart Bryant, Mary Katherine Mills, Bradley JSC Olson, Kristin Michel

**Affiliations:** * Division of Biology, Kansas State University, Manhattan, KS 66506 and; † Department of Biology & Geology, University of South Carolina-Aiken

**Keywords:** miRNA, small RNAs, mosquito reproduction, mosquito tissue, malaria

## Abstract

Malaria continues to be a major global health problem, where disease transmission is deeply linked to the repeated blood feeding nature of the anautogenous mosquito. Given the tight link between blood feeding and disease transmission, understanding basic biology behind mosquito physiology is a requirement for developing effective vector-borne disease control strategies. In the mosquito, numerous loss of function studies with notable phenotypes demonstrate microRNAs (miRNAs) play significant roles in mosquito physiology. While the field appreciates the importance of a handful of miRNAs, we still need global mosquito tissue miRNA transcriptome studies. To address this need, our goal was to determine the miRNA transcriptome for multiple tissues of the pre-vitellogenic mosquito. To this end, by using small RNA-Seq analysis, we determined miRNA transcriptomes in tissues critical for mosquito reproduction and immunity including (i) fat body-abdominal wall enriched tissues, (ii) midguts, (iii) ovaries, and (iv) remaining tissues comprised of the head and thorax. We found numerous examples of miRNAs exhibiting pan-tissue high- or low- expression, tissue exclusion, and tissue enrichment. We also updated and consolidated the miRNA catalog and provided a detailed genome architecture map for the malaria vector, *Anopheles gambiae*. This study aims to build a foundation for future research on how miRNAs and potentially other small RNAs regulate mosquito physiology as it relates to vector-borne disease transmission.

Malaria is a major health problem world-wide, where disease transmission is tightly linked to the repeated blood feeding nature of the anautogenous mosquito, *Anopheles gambiae*. Thus, understanding how the mosquito processes a blood meal for egg production is vital for future vector control strategies. Upon digesting a blood meal, the female mosquito’s physiology drastically changes, with over 50% of her genes showing significant changes in transcript levels (Marinotti *et al.* 2006). While mechanisms are known for how gene expression fluctuates during a blood meal at the transcriptional level (Attardo *et al.* 2005), the mechanisms of post-transcriptional regulation of gene expression are largely unknown. As a much-needed step to address this gap in knowledge, the vector biology field is currently exploring post-transcriptional regulation of mRNA transcripts and its effects on female mosquito physiology by studying a class of small RNAs called microRNAs (miRNAs) (Mead and Tu 2008; Li *et al.* 2009; Gu *et al.* 2013; Hu *et al.* 2015; Skalsky *et al.* 2010; Biryukova *et al.* 2014; Liu *et al.* 2017; Jain *et al.* 2014; Castellano *et al.* 2015; Liu *et al.* 2014b; Jain *et al.* 2015; Allam *et al.* 2016; Lampe and Levashina 2018; Fu *et al.* 2017; Liu *et al.* 2014a; Zhao *et al.* 2017; Zhang *et al.* 2016; Lucas *et al.* 2015a; Lucas *et al.* 2015b; Ling *et al.* 2017; Zhang *et al.* 2017; Akbari *et al.* 2013).

Since the discovery of *lin-14* in 1993 (Lee *et al.* 1993) and the functional characterization of *let-7* in 2000 within *Caenorhabditis elegans* (Reinhart *et al.* 2000), numerous studies in other metazoans have demonstrated that miRNAs regulate developmental timing, drive convergent evolution, and maintain tissue homeostasis (Carthew *et al.* 2017). miRNAs are small non-coding RNA molecules of approximately 22 nucleotides (nt) in length, where the coding region of the miRNA reside either as individual coding sequences or as a cluster, and are either expressed in numerous or single tissues (Carthew *et al.* 2017). Approximately two-thirds of miRNAs are expressed from intergenic regions of the genome. The remaining third of miRNAs are intragenic, mostly found in introns, many of which are thought to be hot spots for novel miRNA evolution (Berezikov 2011). Intragenic miRNAs are conventional miRTrons or 3′ tailed miRTrons, depending on their placement within the intron of the gene (Westholm and Lai 2011). Intergenic miRNAs follow canonical processing using RNA polymerase II for transcription to form a pri-miRNA, Drosha for cleavage to form the pre-miRNA, and Exportin-5 for exportation from the nucleus to the cytoplasm (Liu *et al.* 2008). In contrast, miRTrons do not require Drosha for cleavage (Westholm and Lai 2011). Both intergenic and intragenic miRNAs control mRNA transcript abundance by physically binding the mRNA transcript, resulting in either degradation or translation inhibition (Okamura *et al.* 2007; Liu *et al.* 2008).

Mosquito biologists appreciate miRNAs are important for mosquito physiology relevant to vector-borne disease transmission. Deep sequencing studies in *Aedes albopictus*, *Aedes aegypti*, *Culex quinquefasciatus*, *Anopheles stephensi*, *Anopheles anthropophagus*, *Anopheles funestus*, and *An. gambiae* demonstrate (i) evolutionarily conserved miRNAs, (ii) mosquito-specific miRNAs, and lastly (iii) novel miRNAs (Mead and Tu 2008; Li *et al.* 2009; Gu *et al.* 2013; Hu *et al.* 2015; Skalsky *et al.* 2010; Biryukova *et al.* 2014; Liu *et al.* 2017; Jain *et al.* 2014; Castellano *et al.* 2015; Liu *et al.* 2014b; Jain *et al.* 2015; Allam *et al.* 2016; Akbari *et al.* 2013; Zhang *et al.* 2017). By microarray technology, a recent study in *Anopheles gambiae* found midgut- and ovary-enriched miRNAs (Lampe and Levashina 2018). A recent report found mosquito miRNAs to preferentially load into Argonaute 1 over Argonaute 2 (Fu *et al.* 2017), similar to *Drosophila* (Czech and Hannon 2011). Furthermore, functional studies demonstrate miRNAs play significant roles in mosquito physiology, where *miR-1174* and *miR-275* regulate midgut enzyme levels, *miR-309* regulates ovarian development, *miR-8* regulates wingless signaling in the fat body, *miR-1890* regulates juvenile hormone-controlled enzymes, and *miR-277* regulates lipid metabolism (Liu *et al.* 2014a; Zhao *et al.* 2017; Zhang *et al.* 2016; Fu *et al.* 2017; Lucas *et al.* 2015a; Lucas *et al.* 2015b; Ling *et al.* 2017). Thus, continuing to develop our knowledge on miRNA regulation of mosquito physiology will pay dividends for future efficient mosquito vector control strategies.

While the vector biology field has made significant progress in identifying how some miRNAs regulate mosquito physiology, we still lack an overall understanding for how mosquito tissues vary in miRNA abundance. To this end, our overarching goal was to elucidate how across tissues miRNA transcriptomes in the pre-vitellogenic mosquito are different or similar. In addition, we updated and consolidated the miRNA catalog, provided a miRNA loci genome map, and found certain tissues to possess more multi-mapping small RNAs over other tissues. Altogether, this study provides an essential foundation for furthering our appreciation of the importance of miRNAs and other small RNAs for mosquito physiology in the malaria vector, *An. gambiae*.

## Materials and Methods

### Animals and Tissues

The *An*. *gambiae* G3 strain was reared as previously described (An *et al.* 2011). Adult female mosquitoes were collected at 3-5 days post-eclosion and four tissue groups were dissected. Adult female (i) midgut, (ii) ovaries, (iii) fat body-enriched abdominal walls (fat body-Ab), and (iv) remaining mosquito tissues including head and thorax were obtained. Dissected tissues were stored in DNA/RNA Shield (Zymo Research, Irvine, CA, USA) at 4° until further processing. For each of the four mosquito tissue groups, three separate cages of mosquitoes were used for the three biological replicates.

### Small RNA Library Preparation and Sequencing

Mosquito tissues were removed from DNA-RNA Shield (Zymo Research) and further processed for RNA by using The Direct-zol RNA MiniPrep Kit (Zymo Research) following manufacturers protocol. While Direct-zol removes most DNA, remaining DNA was digested by on-the-column DNAse I treatment. Prior to library construction, RNA quality was assessed with Agilent 2100 Bioanalyzer using an RNA6000 Nano kit chip (Agilent, Santa Clara, CA, USA). For each tissue sample, 1 µg of total RNA was used as input into Illumina TruSeq Small RNA Sample Preparation Kit v2 (Illumina, San Diego, CA, USA). Following library construction, libraries were indexed with Illumina Small RNA adapters, followed by 15 cycle PCR amplification (Illumina). Following library preparation, a Sage Pippin Prep 3% cassette size fractionation system (Sage Science, Inc, Beverly, MA, USA) was used to capture fragment sizes ranging from 125-160bp, which includes the 113bp adapter sequence. The Sage Pippin Prep cassette size capture system targets small RNAs approximately within 13 - 48bp. To validate purified libraries, the Agilent 2100 Bioanalyzer was used with the High Sensitivity DNA Kit (Agilent, Santa Clara, CA, USA). Following quality control of library preparation and subsequent library quantification, small RNA libraries were adjusted to 2 nM and pooled for multiplexed sequencing. The small RNA libraries were sequenced for 50 cycles on a HiSeq2500 in Rapid Read Run using the TruSeq Rapid SBS kit-HS (Illumina). Sequence data were converted to FASTQ files and de-multiplexed into individual sequences for further downstream analysis.

### Small RNA Sequence Analysis

Analysis of FASTQ files obtained from the small RNA libraries was performed using CLC Genomics Workbench version 11.0.1 (Qiagen, Hilden, Germany). Raw reads from 12 libraries, composed of four mosquito tissue types with three biological replicates, were trimmed of adapter sequences. For miRNA analysis, trimmed sequences were size filtered for reads of 20 - 24nt in length. Trimmed and size-selected reads were mapped to the *An. gambiae* PEST genome, AgamP4 (Giraldo-Calderón *et al.* 2015). Next, mapped reads were divided into multi-mapping- or unique- reads. As multi-mapping reads are repetitive in nature, only their percentage from the total reads was determined. Further, the multi-mapping reads were removed from further analysis, routine practice for miRNA transcriptomic studies (Sherstyuk *et al.* 2017; Wen *et al.* 2014). Unique reads were queried against miRBase v22 (Kozomara and Griffiths-Jones 2014) pre-miRNA sequences from *An. gambiae* and *Ae. aegypti* mosquitoes and miRNAs from recent studies (Fu *et al.* 2017; Biryukova *et al.* 2014), allowing up to 2nt mismatches. Reads matching these sequences were designated as annotated reads. Reads not matching these sequences were designated as unannotated reads. For annotated reads, each miRNA was manually inspected to ensure accuracy of annotated miRNA read counts determined by CLC Workbench. For unannotated reads, these sequences were fed into miRDeep2 with default parameters (Friedländer *et al.* 2012) as a means to determine candidate miRNAs. Predicted candidate miRNAs were examined manually to assess their potential based on guidelines suggestive of RNase III enzymatic cleavage (Wen *et al.* 2014; Berezikov *et al.* 2011): (1) candidate sequence must form a stable hairpin structure of at least -30kcal/mol using RNAfold (Gruber *et al.* 2008), (2) candidate sequence must have at least one star strand read, and (3) the 5p and 3p miRNA sequence must pair with at least a 2-nt overhang. To account for redundancy of miRNA nomenclature, all candidate miRNAs were cross-referenced to miRNAs reported in (Fu *et al.* 2017; Biryukova *et al.* 2014), which are not currently represented in v22 miRBase. Finally, candidate miRNAs were then included in the annotated read group for chromosome mapping analysis.

For miRNA genome map illustration purposes, chromosomal locations of miRNAs obtained from VectorBase through BLAST (Giraldo-Calderón *et al.* 2015) were constructed with Adobe Illustrator (Adobe Systems, San Jose, CA, USA) using a 10,000 bp:1 pixel ratio. For miRNA expression analysis, all miRNAs were interrogated for expression in our twelve small RNA-Seq libraries with annotated reads converted to reads per million (RPM). Normalized log_10_ miRNA expression values were used to generate a heatmap using Morpheus from the Broad Institute (software.broadinstitute.org/morpheus/). Values were hierarchically clustered by Euclidean distance for the metric, average for linkage method, and rows (miRNAs) and columns (tissues) were clustered. The heatmap for the miRNA cluster expression analysis used the relative color scheme option in Morpheus (software.broadinstitute.org/morpheus/), which assesses the minimum and maximum values for each miRNA across tissues and converts the expression data into colors for strictly qualitative non-statistical assessment purposes. Principle component analysis (PCA) was performed on annotated reads RPMs across tissues with singular value decomposition (SVD) along with no scaling as a means to keep the original variability of the data (Metsalu and Vilo 2015). Qualitative guidelines for categorization of miRNA tissue expression patterns include the following. miRNAs with expression values ≥3.5 log_10_ RPM across all tissues were designated pan-tissue high expression. miRNAs with expression values ≤2.0 log_10_ RPM across all tissues were designated pan-tissue low expression. miRNAs with tissue exclusion or tissue enrichment were determined by comparing the average expression values between the two lowest and highest expressing tissues for each miRNA, respectively. miRNAs with ≥0.5 log_10_ RPM difference between the two lowest tissues had a tissue exclusion expression pattern. Conversely, miRNAs with ≥0.5 log_10_ RPM difference between the two highest had a tissue enrichment expression pattern.

### Quantitative PCR

Quantification of miRNA expression was performed as previously demonstrated (Bryant *et al.* 2010). RNA was extracted with same methods described for small RNA libraries. Reverse transcription (RT) was performed using Qiagen miScript II RT Kit (Qiagen) followed by quantitative PCR (RT-qPCR) using the Qiagen miScript SYBR Green PCR Kit (Qiagen). The PCR condition was as follows: Step 1, 95° 15 min; Step 2, 95° for 15 s, 56° for 30 s, 70° for 30 s for 40 cycles; Step 3, 95° 1 min; melt curve analysis. For miRNA expression, (i) forward primers were the sequence of the mature miRNA up to 58° Tm, where miRNA candidates with a low Tm contained extra sequence added to the 5′ end of the forward primer, and (ii) the reverse primer for all miRNAs was the universal reverse primer, as described previously (Bryant *et al.* 2010). All primer sequences are listed in Table S1. Ribosomal *S7* gene served as the normalizer. Transcript expression was determined by 2^-∆Ct^ (Schmittgen and Livak 2008).

## Data Availability

Sequencing data have been submitted to NCBI SRA database (ncbi.nlm.nih.gov/sra) under accession number PRJNA435430. Data were also submitted to Vectorbase (Giraldo-Calderón *et al.* 2015). Supplemental material available at Figshare: https://doi.org/10.25387/g3.7732994.

## Results

### Small RNA sequencing of An. gambiae tissues

**Figure 1:**
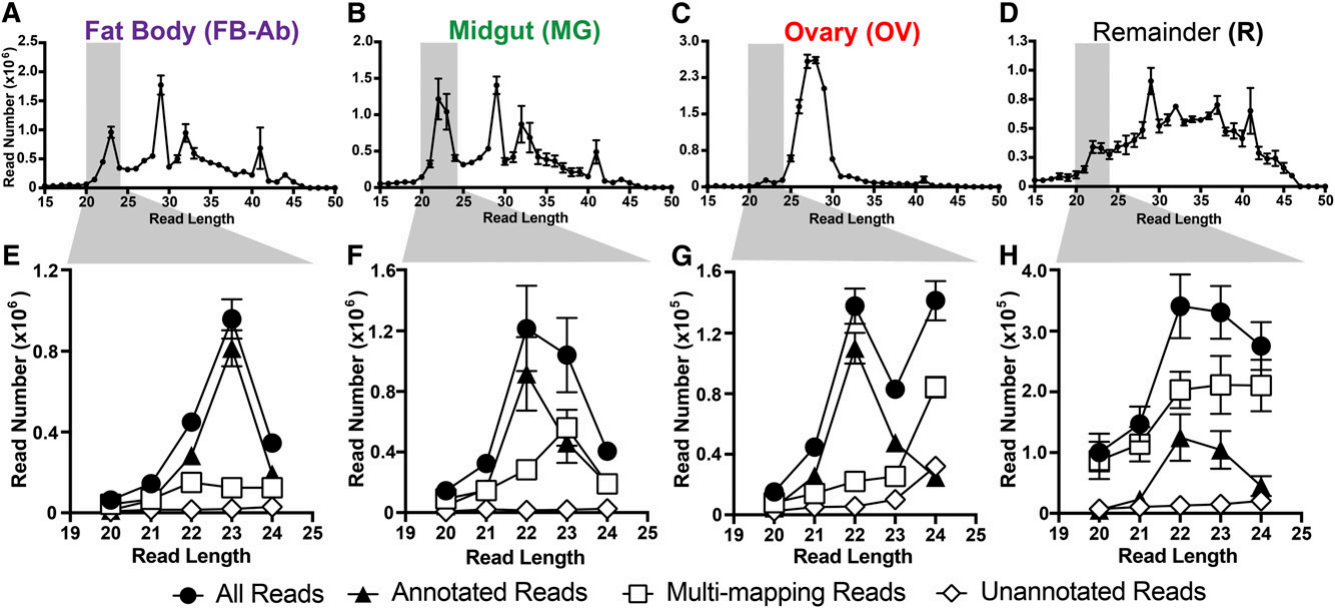
Summary of small RNAs in *An. gambiae* mosquito tissues. Female mosquitoes were divided into differing tissue groups, fat body-abdominal walls (FB-Ab, purple), midgut (MG, green), ovary (OV, red), and remaining tissues comprised of mosquito head and thorax (R, black). For size distributions of small RNAs across mosquito tissue groups, (A-D) histograms of read length over read number show the diverse small RNA read distributions for each mosquito tissue group. For each tissue group, size-selected small RNAs of 20-24 nts highlighted in gray were put into four classifications (E-H): (1) All Reads, designated with black circles, represented total reads, (2) Annotated Reads, designated with black triangles, represented annotated miRNAs, (3) Multi-mapping Reads, designated with white squares, represented non-unique sequence reads, and (4) Unannotated Reads, designated with white diamonds, represented undetermined small RNAs. All values represent three biological replicates, and values are graphed as average +/− SEM.

To determine the miRNA transcriptome at the tissue level for *An. gambiae*, we divided the female mosquito into four tissue groups, (i) abdominal walls for fat body-Ab sample, (ii) midguts, (iii) ovaries, and (iv) remaining tissues comprised of the head and thorax. From these four tissue groups, each with three biological replicates, we generated and sequenced twelve small RNA libraries, which resulted in a total 148.2 million reads. Reads trimmed of adaptor sequences yielded a total of 141.3 million reads (Table S2). Across all tissues, the number of trimmed reads was roughly similar with a range of 10.8-12.3 million reads (Table S2). However, size profile distributions of these small RNA reads demonstrated a noticeable difference across tissues (Figure 1A-D). Fat body-Ab and midgut tissues possessed peaks at 22-23, 29, 32, and 41nts (Figure 1A and B), ovary tissue possessed peaks mostly from 25-30nts (Figure 1C), and remainder tissue only yielded a slight peak at 29nt (Figure 1D).

To focus on miRNAs, we size-filtered for reads within 20-24nts. Here, we obtained a total of 20.1 million reads across all tissue groups (Figure 1E-H). On average for these size-filtered small RNAs: the fat body-Ab had ∼1.9 million reads, the midgut had ∼3.1 million reads, the ovary had ∼0.4 million reads, and the remainder had ∼1.2 million reads (Table S2). The size-filtered reads mapped to the genome with a range of 99.4–99.7% (Table S2). Further, these reads were partitioned into four groups based on two parameters, (i) genome mapping properties and (ii) miRNA gene annotation (explained in Materials and Methods) (Figure 1E-H). The first group, all reads, simply contained all reads and represented the sum of the remaining three groups discussed below. The second group, annotated reads, contained reads with unique mapping properties and miRNA gene annotation based on miRBase databases, miRNAs reported in (Fu *et al.* 2017; Biryukova *et al.* 2014), and miRDeep2 candidate miRNAs. The third group, multi-mapping reads, contained reads with non-unique mapping properties with no annotation. The fourth group, unannotated reads, contained reads with unique mapping properties but no available annotation.

The unannotated group contained the smallest number of reads representing ∼6.5% of size-filtered small RNA reads across all tissues (Figure 1E-H and Table S2), where small RNAs species within this group remain to be discovered in future studies. Interestingly, on average across all tissues ∼43.3% of the size-filtered small RNA reads were multi-mapping (Figure 1E-H and Table S2), in agreement with other mosquito small RNA studies demonstrating that close to half of small RNA reads are multi-mapping (repetitive or non-unique) (Fu *et al.* 2017; Biryukova *et al.* 2014). Furthermore, each tissue group varied in the percentage of multi-mapping small RNAs. On average, multi-mapping reads accounted for ∼26.2% for the fat body-Ab, ∼41.8% for the midgut, ∼36.6% for the ovary, and ∼68.6% for remaining tissues of the size-filtered small RNAs (Figure 1E-H and Table S2). The annotated group represented the majority of size-filtered small RNA reads at ∼50.2% across all tissues (Figure 1E-H and Table S2), and were the focus of the study. For the rest of the results, only annotated reads are discussed.

### Annotated miRNA systematics

Our small RNA libraries yielded a catalog of 139 unique miRNAs (complete catalog found in Dataset S1) comprised of 103 *An. gambiae* v22 miRBase sequences, 8 *Ae. aegypti* orthologs, 21 previously reported novel miRNAs (Fu *et al.* 2017; Biryukova *et al.* 2014), and 7 candidate miRNAs predicted by miRDeep2 in this study. For v22 miRBase Anopheles sequences, we found 103 miRNAs represented in our libraries and removed 26 v22 miRBase Anopheles sequences due to their genome multi-mapping properties. Removing multi-mapping small RNAs is a common practice in miRNA transcriptomic studies (Sherstyuk *et al.* 2017; Wen *et al.* 2014; Biryukova *et al.* 2014; Fu *et al.* 2017) (Dataset S2). For *Ae. aegypti* orthologs, our libraries contained both previously reported miRNAs *miR-71*, *miR-252*, *miR-999*, and *miR-2940* (Biryukova *et al.* 2014) and miRNAs discovered in this current study, *miR*-193, *miR-316*, *miR-2765*, and *miR-2942* (Dataset S1). For previously reported 41 novel miRNAs (Fu *et al.* 2017; Biryukova *et al.* 2014), our libraries possessed 21 of these miRNAs (Dataset S1) and 20 of these novel miRNAs (Fu *et al.* 2017; Biryukova *et al.* 2014) were removed due to (i) genome multi-mapping properties or (ii) redundant miRNA naming (Sherstyuk *et al.* 2017; Wen *et al.* 2014; Biryukova *et al.* 2014; Fu *et al.* 2017) (Dataset S2). For candidate miRNAs in this study, unannotated mapped reads were further interrogated by miRDeep2 (Friedländer *et al.* 2012) where output yielded (1) a predicted hairpin, (2) miRNA -star and -mature sequences, (3) number of reads for candidate miRNA, (4) and number of mismatch reads for candidate miRNA. Here, we obtained 7 candidate miRNAs, and kept the generic miRDeep2 ID, as future work is needed to determine their importance in mosquito physiology (Dataset S1 and Figure S2).

### Genome mapping properties for annotated miRNA reads

**Figure 2:**
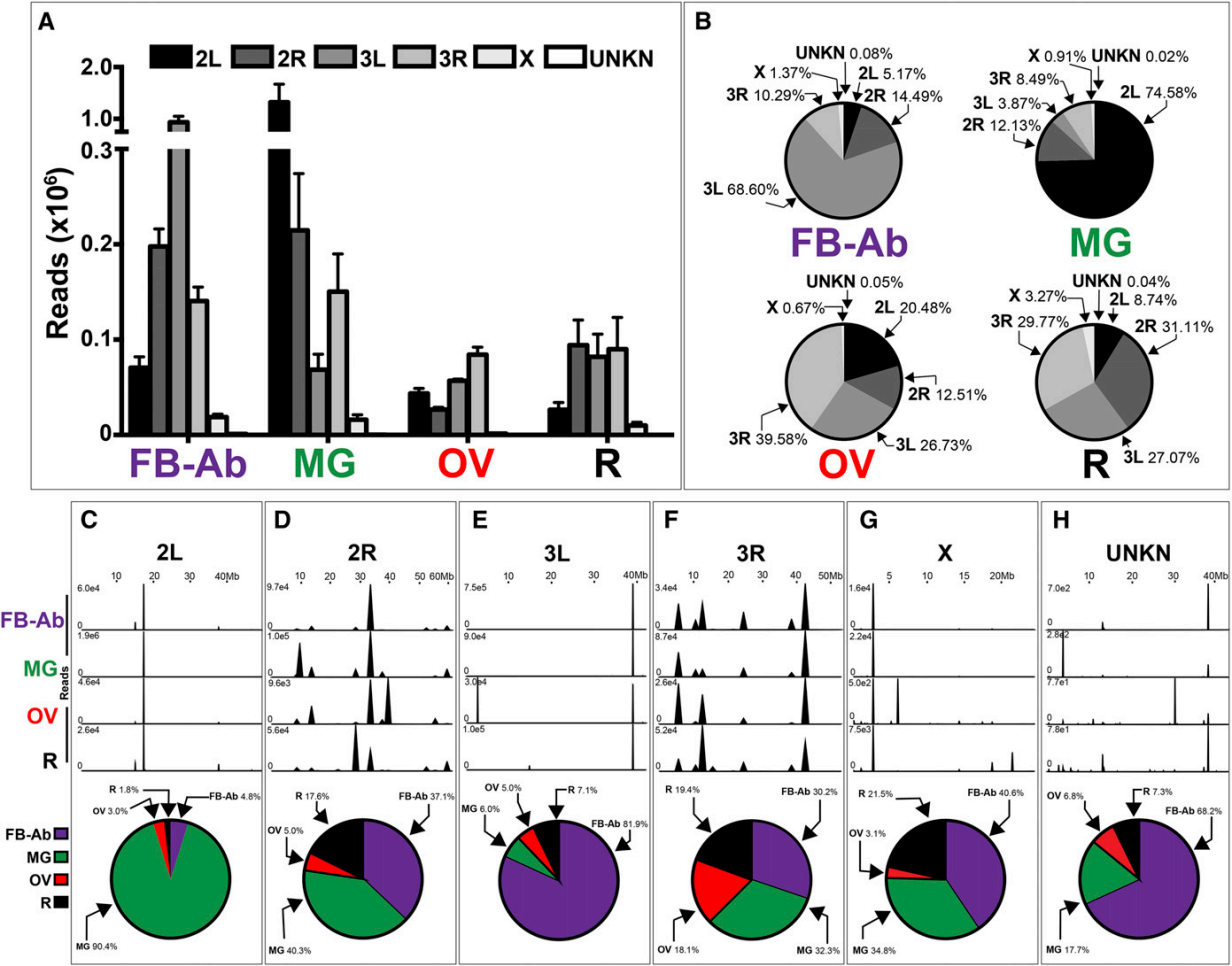
Genome-wide mapping of annotated reads in *An. gambiae* mosquito tissues. (A-B) Chromosomal contribution of annotated reads to each tissue miRNA transcriptome. Data are graphed as (A) annotated read count and (B) percentage of annotated reads. (C-H) miRNA expression hot spots across chromosomes, (C) 2L, (D) 2R, (E) 3L, (F) 3R, (G) X, and (H) UNKN, for each tissue group, which include the percentage of tissue contribution to mapped annotated reads as graphed by pie graphs. Mosquito tissues include the Fat body-abdominal walls (FB-Ab, purple), Midgut (MG, green), Ovary (OV, red), and Remainder (R, black). This figure shows a representative from three biological replicates, with additional replicates available in (Figure S3). (A) values represent three biological replicates, and values are graphed as average +/− SEM. (B, C-H) Average values from three biological replicates were used for pie graphs.

As our main goal was to determine the uniqueness of the various mosquito tissue miRNA transcriptomes, (i) we mapped annotated reads across all chromosomes to determine the chromosomal contribution for each tissue’s miRNA transcriptome, and (ii) for each chromosome, determined genome placement and the level of annotated read intensity across all tissues. However, it is important to note that the number of annotated reads vary for each tissue group. For example, while on average the fat body-Ab and midgut had ∼1.4 million and ∼1.8 million annotated reads, respectively (Table S2), ovary and remainder tissues had a lower number of annotated reads at ∼0.2 and ∼0.3 million reads, respectively (Table S2). Both fat body-Ab and midgut possessed more annotated reads mapped to individual chromosomes compared to ovary and remaining tissues (Figure 2A).

By determining the percentage of mapped annotated reads across chromosomes (Figure 2B), we determined the chromosomal contribution for each mosquito tissue miRNA transcriptome. We found chromosomes 2L, 2R, 3L, and 3R contributed roughly similar to ovary- and remainder- tissues’ miRNA transcriptomes with a range of 12.51–39.58% and 8.74–31.11%, respectively (Figure 2B). Conversely, for fat body-Ab and midgut tissues, chromosome 3L at 68.60% and chromosome 2L at 74.58% contributed the most to these tissue miRNA transcriptomes, respectively (Figure 2B). Across all tissues, X and UNKN chromosomes contributed minimally with highest values only at 3.27% for the remainder tissue and 0.08% for the fat body-Ab, respectively (Figure 2A-B).

At the chromosomal level with varying annotated read intensities across all tissues, we found multiple mapping hot spots (Figure 2C-H). As the number of annotated reads mapped to each chromosome varies for each tissue, it is important to pair mapping data (Figure 2C-H) with total number of reads per tissue (Figure 2A). For example, on chromosome 2L, a majority of the annotated reads mapped to the ∼18Mb locus across all tissues (Figure 2C). However, the midgut contributed 90.4% to these 2L chromosome mapped annotated reads across all tissues (Figure 2C). Similarly, on chromosome 3L, a major portion of the annotated reads across tissues mapped to the ∼39Mb locus across all tissues, but the fat body-Ab contributed 81.9% to these 3L chromosome mapped annotated reads across all tissues (Figure 2E). Lastly, while 2R and X chromosomes shared similar trends of tissue contribution to annotated reads mapped to these chromosomes (compare Figure 2D and G), it is important to note that 2R chromosome contributed more to the mosquito’s overall miRNA transcriptome over the X chromosome (Figure 2A-B).

### miRNA genome architecture

**Figure 3:**
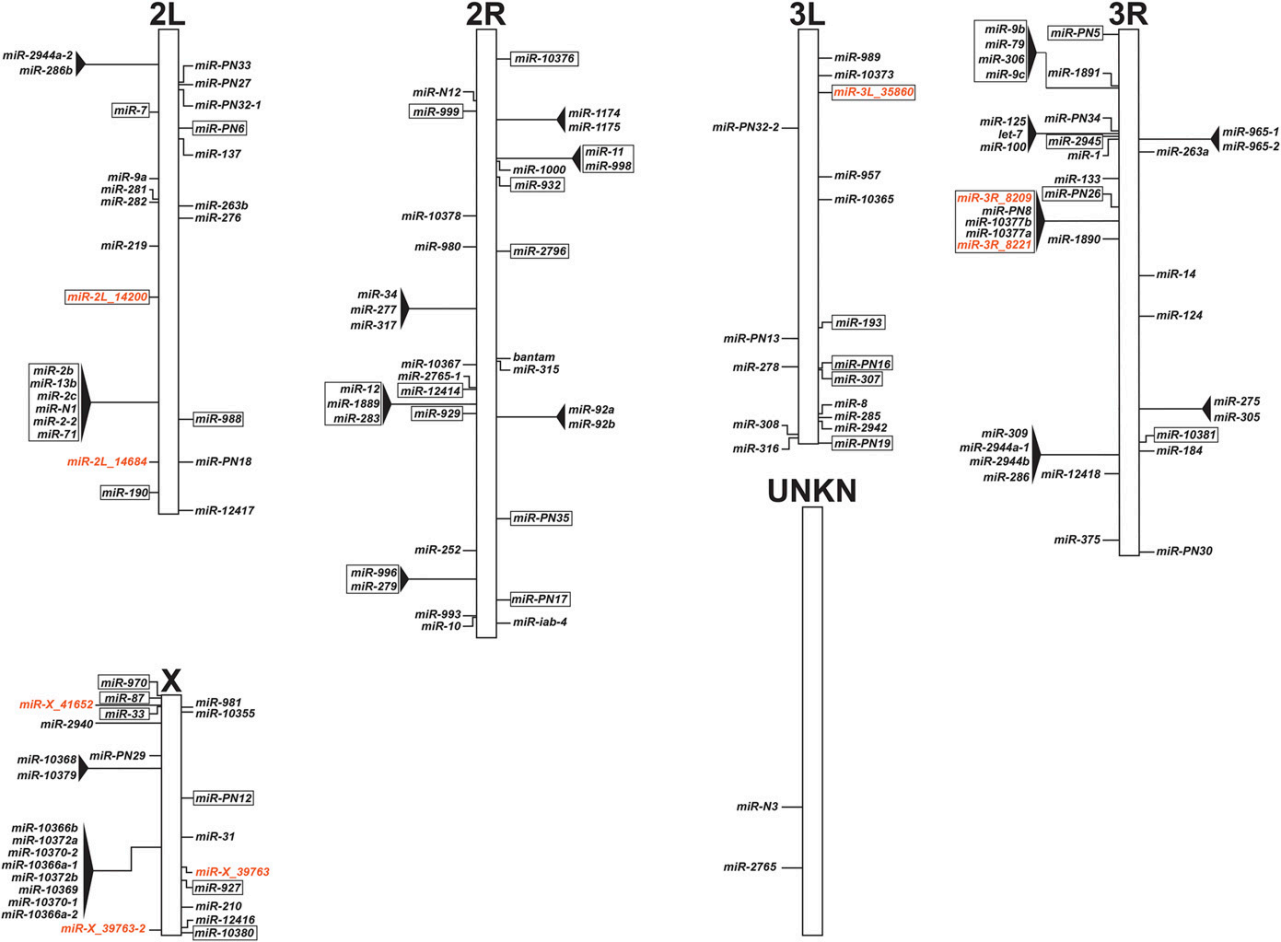
*An. gambiae* miRNA genome map. The distribution of 139 miRNA genes over the *An. gambiae* genome in top-down tier order, with (+) strand annotations on the right of the chromosome and (-) strand annotations on the left of the chromosome. miRNA clusters are represented by black triangles. Intragenic miRNAs, which reside within genes, are represented by individual box outlines. Lastly, intragenic miRNA clusters are represented by black triangles and an all-encompassing box outline. Current annotated miRNAs are in black, while candidate miRNAs from this study are in orange.

In addition to updating and consolidating the *An. gambiae* miRNA catalog (Datasets S1 thru S3), we further developed a concise miRNA genome architecture map for future work on *An. gambiae* miRNAs. The miRNA genome architecture map illustrates the *An. gambiae* chromosome placement for all 139 miRNAs (Figure 3), further corresponding datasets are found in (Datasets S1 and S3). The miRNA genome architecture map highlights intergenic- and intragenic- miRNAs, as well as clusters for these two different miRNA types (Figure 3). Similar to previous studies, 64.0% of the miRNAs were intergenic, while the remaining 36.0% were intragenic (Westholm and Lai 2011; Rodriguez *et al.* 2004; Biryukova *et al.* 2014). For the intragenic miRNAs, 88.0% were intronic, 6.0% were conventional miRTrons, 4.0% were 3′ tailed miRTrons, and 2.0% were within an exon (Dataset S3). Corresponding *Anopheles gambiae* Pest strain (AGAP) numbers for these miRNAs can be found in Datasets S1 and S3. Based on previous studies, groups of miRNAs were designated as clusters if they resided within 10 kb (Marco *et al.* 2013; Mohammed *et al.* 2014). Indeed, 37.4% of miRNAs resided in clusters, of which we found conserved miRNA clusters (Marco *et al.* 2013; Liu *et al.* 2014a) and novel miRNA clusters comprised of our miRDeep2 candidate miRNAs and previously described miRNAs (Fu *et al.* 2017; Biryukova *et al.* 2014) (Dataset S3). Lastly, 42.3% of miRNA clusters were intragenic (Figure 3 and Dataset S3).

By combining the genome mapping properties of annotated reads (Figure 2) with the miRNA genome architecture map (Figure 3), we correlated mapping annotated read peaks to a single miRNA or multiple miRNAs. For example, on chromosome 2L across all tissues, a prominent peak of mapped reads at approximately 18Mb (Figure 2C) represented *miR-281* (Figure 3). On chromosome 3L across all tissues, a prominent peak of mapped reads at approximately 39Mb (Figure 2E) represented *miR-8* (Figure 3). On chromosome 3L in ovary tissue, a prominent peak at approximately 3Mb (Figure 2E) represented *miR-989* (Figure 3). Lastly, on chromosome 2R in ovary tissue, a prominent peak at approximately 39Mb (Figure 2D) represented *miR-92a* and *miR-92b* (Figure 3). Altogether, these prominent specific miRNAs accounted for a substantial proportion of the mapped annotated reads.

### Multiple tissue miRNA transcriptome-wide analysis in An. gambiae

**Figure 4:**
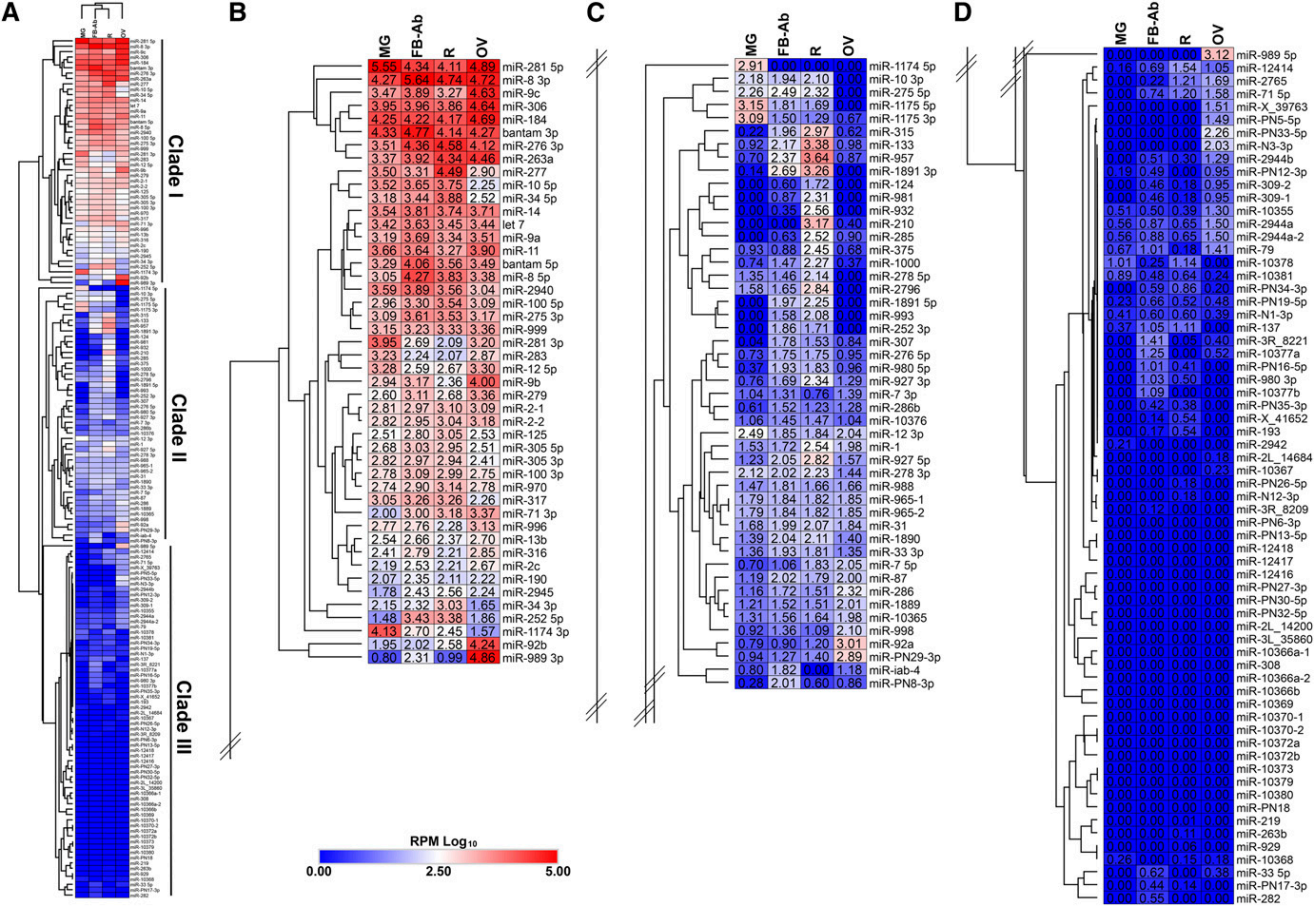
Mosquito tissue miRNA transcriptome. (A) Heatmap of 139 miRNA genes across mosquito tissues. Mosquito tissues include the Fat body-abdominal walls (FB-Ab), Midgut (MG), Ovary (OV), and Remainder (R). Expression levels were graphed as log_10_ transformed average reads per million (RPM) from three biological replicate libraries. Heatmap was separated into three clades based on overall expression with (B) Clade I representing highest expressed miRNAs, (C) Clade II representing middle, and (D) Clade III representing lowest expressed miRNAs.

First, PCA was performed on annotated reads RPM across tissues, where biological replicates for each tissue sufficiently grouped (Figure S1). Further, to illustrate miRNA expression across mosquito tissue groups, reads were converted to RPM and the average for the three biological replicates were log_10_ transformed (Dataset S4). Dendrograms of the different tissue groups illustrated fat body-Ab and remainder tissues clustered closely together, followed by midgut and ovary tissues (Figure 4A, top of heatmap). To illustrate the range of miRNA expression across tissues, we separated the heatmap into three clades, Clade I representing the highest expressed miRNAs (Figure 4B), Clade II representing middle (Figure 4C), and Clade III representing lowest expressed miRNAs (Figure 4D). To validate our mosquito tissue miRNA transcriptome data, fourteen miRNAs were assessed for tissue expression trends by RT-qPCR analysis. In all cases, miRNA tissue expression trends as determined by RT-qPCR agreed with miRNA tissue expression trends as determined by small RNA-Seq (Figure S4).

**Figure 5:**
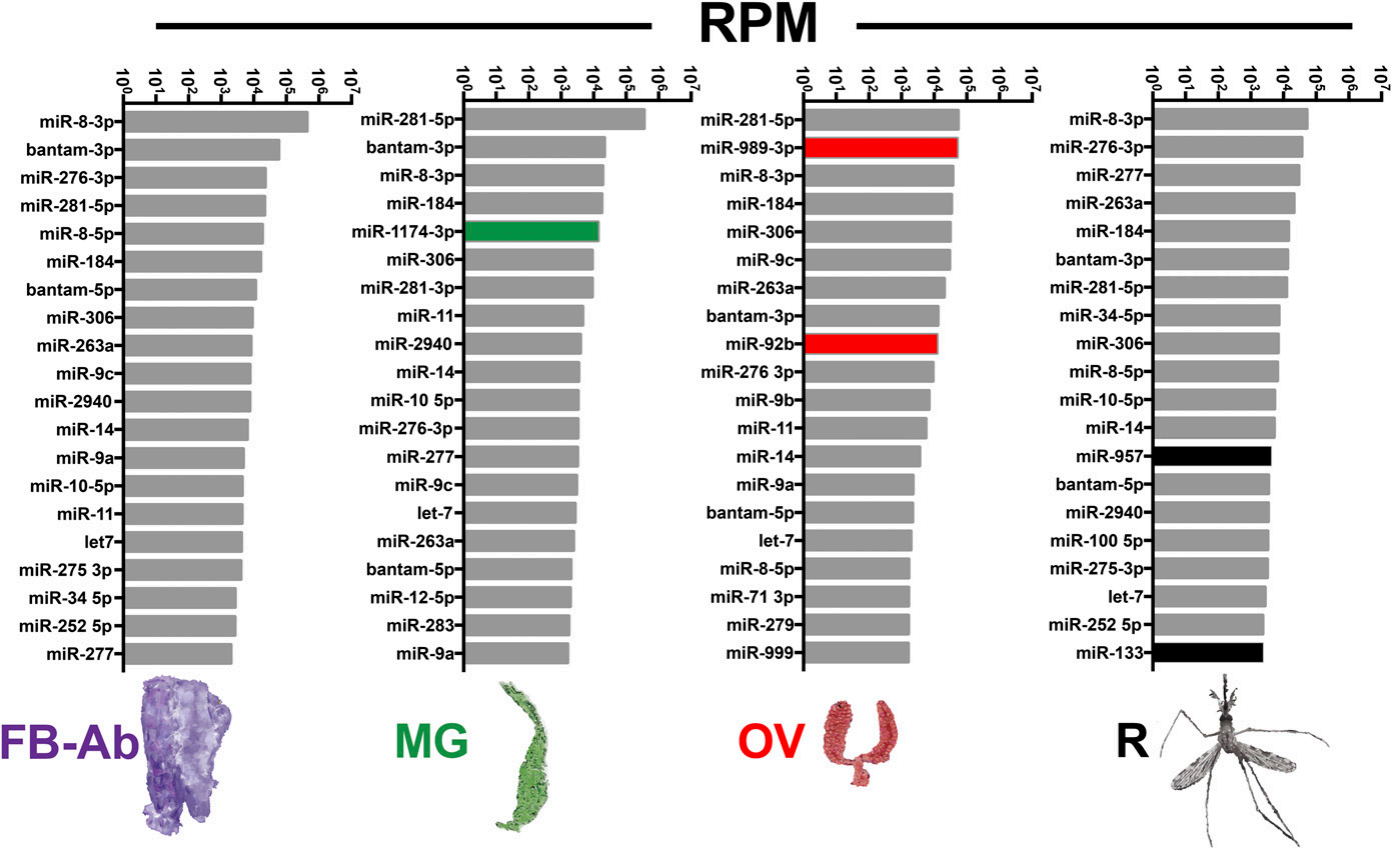
Most abundant miRNAs in differing mosquito tissue groups. The top twenty miRNAs for each tissue small RNA-Seq library. Values graphed are average reads per million (RPM) from three biological replicates. Mosquito tissues include the Fat body-abdominal walls (FB-Ab, purple), Midgut (MG, green), Ovary (OV, red), and Remainder (R, black). Tissue enriched miRNAs, *miR-1174* for MG, *miR-989* and *miR-92b* for OV, and *miR-957* and *miR-133* for R, are highlighted in their respective small RNA-Seq library tissue group.

Our small RNA-Seq analysis found a myriad of miRNA tissue expression patterns for most but not all miRNAs. To determine miRNA tissue expression patterns, miRNA expression values were interrogated with a qualitative set of guidelines to yield (i) pan-tissue high expression, (ii) pan-tissue low expression, (iii) tissue exclusion, and (iv) tissue enrichment (see Methods for guidelines, see Dataset S5 for miRNA list). Of important note, there were varying degrees of tissue -exclusion or -enrichment across all miRNAs. Examples of miRNAs with pan-tissue high expression included *miR-281*, *miR-8-3p*, *miR-306*, *miR-184*, *bantam*, *miR-276*, and *miR-14* which resided within Clade I (Figure 4B) and within the top twenty most abundant miRNAs across all tissues (Figure 5). On the converse, the majority of the miRNAs exhibited pan-tissue low expression (Dataset S5), some within Clade II but mostly within Clade III (Figure 4C, D). Examples of miRNAs excluded from ovary include *miR-10*, *miR-34*, *miR-2940*, and *miR*-317 (Dataset S5) within Clade I (Figures 4B). Examples of miRNAs excluded from midgut include *miR-276 3p*, *miR-263a*, and *miR-71* (Dataset S5) within Clade I (Figure 4B). Examples of miRNAs excluded from remainder tissue include *miR-281 3p* and *miR-9b* (Dataset S5) within Clade I (Figure 4B).

We found multiple examples of miRNAs with tissue enrichment for each tissue type analyzed with certain miRNAs demonstrating higher degrees of tissue enrichment over others. For example, while *miR-8-3p* was in the top twenty miRNAs across all tissues (Figure 5), it was enriched in fat body-Ab tissue (Dataset S5) within Clade I (Figure 4B). Additionally, *miR-PN8* from (Fu *et al.* 2017; Biryukova *et al.* 2014) exhibited slight enrichment in fat body-Ab tissue (Dataset S5) within Clade II (Figure 4C). Similarly, even though *miR-281* belonged to pan-tissue high expression (Dataset S5) and resided in the top twenty miRNAs across tissues (Figure 5), this miRNA was enriched in midgut tissue (Dataset S5) within Clade I (Figure 4B). Additionally, *miR-1174* and *miR-1175* were enriched in midgut tissues (Dataset S5) within Clades I and II, respectively, agreeing with previous reports (Liu *et al.* 2014a; Lampe and Levashina 2018) (Figure 4B-C and Figure 5). Examples of miRNAs enriched in remainder tissue included *miR-957* and *miR-133* from Clade II (Figure 4B-C, Figure 5, Dataset S5). Examples of miRNAs enriched in ovary tissue included *miR-989* (Clade I), *miR-92b* (Clade I), *miR-92a* (Clade II), *miR-PN29* (Clade II), *miR-PN5* (Clade III), *miR-PN33* (Clade III), *miR-N3* (Clade III), *miR-2944b* (Clade III), and *miR-309* (Clade III) with varying degrees of ovary tissue enrichment (Figure 4B-D and Dataset S5). Of interest, *miR-PN8*, *miR-PN29*, *miR-PN5*, *miR-PN33*, and *miR-N3*, (Fu *et al.* 2017; Biryukova *et al.* 2014) are not represented in v22 miRBase for Anopheles. Future work is warranted to determine which of these tissue-enriched miRNAs retain or lose their tissue expression patterns after stimuli such as blood feeding or plasmodium infection.

Hierarchical clustering of all miRNA expression data did not find any of the miRNAs within their respective cluster to exhibit co-expression patterns across tissues (Figure 4). However, by analyzing miRNA clusters alone with the ‘relative color scheme’ option in Morpheus (see Methods) for strictly qualitative non-statistical assessment, nine of the sixteen miRNA clusters demonstrated co-expression patterns within their respective cluster across tissues. Examples included: (i) *miR-1174* and *miR-1175* on chromosome 2R, (ii) miR-92a and miR-92b on chromosome 2R, and (iii) *miR-309*, *miR-2944a-1*, *miR-2944b*, and *miR-286* on chromosome 3R (Figure S5).

## Discussion

By small RNA-Seq analysis, we determined the diverse range of small RNAs across *An. gambiae* mosquito tissues. This study (i) updated and consolidated the malaria vector miRNA catalog with additional Aedes orthologs and seven candidate miRNAs, (ii) made a genome architecture map to highlight chromosome loci for intragenic- and intergenic- miRNAs and their respective clusters, (iii) found mosquito tissues to vary in amount of multi-mapping small RNAs, and (iv) determined assorted miRNA tissue expression patterns in the malaria vector, *An. gambiae*.

A recent report used microarray technology to determine miRNA abundance in mosquito tissues in the *An. gambiae* strain, *An. coluzzi* Ngousso (TEP1*S1) (Lampe and Levashina 2018). Despite differences in mosquito strains and technology, there are numerous examples of where data from both studies agree. For example, both studies found *miR-989* and *miR-1175* to be ovary- and midgut- enriched, respectively (Lampe and Levashina 2018). Additionally, both studies found miRNAs *miR-8*, *miR-306*, and *miR-184* to be highly expressed across tissues (Lampe and Levashina 2018). However, we did find additional data from our small RNA-Seq libraries. Our data found *miR-1174* to be expressed higher than its miRNA cluster companion *miR-1175*, which agrees with *Ae. aegypti* (Liu *et al.* 2014a) but disagrees with (Lampe and Levashina 2018). We found *bantam*, *miR-276*, *miR-263a*, and *miR-14* to be of the top twenty highest miRNAs across all tissues, which disagrees with (Lampe and Levashina 2018) who reported these miRNAs to be lowly expressed across tissues. RNA-seq has many advantages over microarray technology: (i) lower false discovery rate, (ii) lower background noise, and (iii) a larger dynamic range to quantify gene expression (Wang *et al.* 2009; Nault *et al.* 2015), which can explain the data variability between these studies. However, despite these differences, taken together both the (Lampe and Levashina 2018) and this current RNA-seq study, further drive our appreciation and understanding of miRNA differences across mosquito tissues in the malaria vector, *An. gambiae*.

Additionally, our mosquito miRNA transcriptome analysis found multiple miRNAs to exhibit either global tissue expression, tissue exclusion, or tissue enrichment. miRNAs with strong enrichment in a specific tissue are termed tissue-specific miRNAs, where current hypothesis suggests these small RNAs regulate the development of novel cell types as well as maintain tissue homeostasis (Wheeler *et al.* 2009; Christodoulou *et al.* 2010; Stark *et al.* 2005; Chen *et al.* 2004). Examples include, (i) mouse thymus-specific *miR-181*, which regulates hematopoietic tissue (Chen *et al.* 2004), (ii) fruit fly muscle-specific *miR-1*, which regulates muscle physiology (Sokol and Ambros 2005), (iii) mosquito midgut-specific *miR-1174*, which regulates blood digestion enzymes (Liu *et al.* 2014a), and (iv) mosquito ovary-specific *miR-309*, which regulates egg development (Zhang *et al.* 2016). In this study, we found miRNAs enriched in specific tissues to varying degrees. Given the drastic transcriptional changes induced by blood feeding (Marinotti *et al.* 2006), future research is needed to decipher which of these tissue-enriched miRNAs retain or lose their specific tissue expression. Of interest to mosquito reproduction is *miR-989*. This miRNA was exclusively expressed in the ovary, which is in agreement with other mosquito studies (Lampe and Levashina 2018; Mead and Tu 2008; Castellano *et al.* 2015; Jain *et al.* 2015) and the butterfly (*Pararge aegeria*) (Quah *et al.* 2015). In *Drosophila*, functional data demonstrate *miR-989* controls border cell migration within the ovary (Kugler *et al.* 2013), where defects in border cell migration severely affect egg fertilization and result in sterility phenotypes (Montell *et al.* 2012). Given the ongoing interests in mosquito sterility for vector population control strategies (Catteruccia *et al.* 2009), future work on how the ovary-specific *miR-989* regulates the mosquito gonadotrophic cycle should prove fruitful.

A curious part of our mosquito tissue small RNA-Seq data are the substantial number of multi-mapping small RNAs. While common in small RNA-Seq studies (Sherstyuk *et al.* 2017; Wen *et al.* 2014; Johnson *et al.* 2016; Biryukova *et al.* 2014; Fu *et al.* 2017), the current practice is to remove multi-mapping reads from miRNA analysis (Sherstyuk *et al.* 2017; Wen *et al.* 2014). Indeed, here we removed many Anopheles v22 miRBase and previously reported miRNAs (Fu *et al.* 2017; Biryukova *et al.* 2014) due to their multi-mapping nature. However, while we removed these small RNA sequences from the *An. gambiae* miRNA catalog, we are not suggesting they are not important for mosquito physiology. Rather, we suggest they were simply miscategorized. Indeed, some small RNA researchers suggest multi-mapping (or repetitive) small RNAs must regulate some aspect of physiology given their substantial abundance in plants and animals (Johnson *et al.* 2016; Axtell 2013; Slotkin 2018). Reproduction studies in the fruit fly find multi-mapping small RNAs, called repeat associated siRNAs (rasiRNAs), to regulate genomic stability, DNA damage in the germline, embryonic axis specification, and mRNA localization during oogenesis (Theurkauf *et al.* 2006; Vagin *et al.* 2006; Klattenhoff *et al.* 2007). Also, mutant flies lacking the ability to produce rasiRNAs result in sterile females with oogenesis defects (Pane *et al.* 2007). Interestingly, our data show the fat body-Ab and remainder tissues to greatly vary in their abundance of multi-mapping small RNAs, while dendrogram analysis of annotated reads grouped these mosquito tissues together. While the above mentioned small RNAs differ in length to rasiRNAs, 20-24nt *vs.* 25-29nt, respectively, both small RNA groups are multi-mapping. Given the vast abundance of multi-mapping small RNAs across mosquito tissues and the inherent challenges of studying them (Johnson *et al.* 2016; Axtell 2013), future work is needed to decipher their multiple genomic loci, as well as their importance in mosquito physiology. Lastly, this study focused on miRNAs, which account for less than 20% of all small RNAs obtained in small RNA-seq studies, in agreement with other studies performed in mosquitoes (Biryukova *et al.* 2014; Fu *et al.* 2017; Akbari *et al.* 2013). Thus, future work is also needed to classify, categorize, and quantify small RNAs outside the 20-24nt range.

Our tissue miRNA expression data represents the resting reproductive state of the previtellogenic adult female mosquito (Clements 2000). Interestingly, we found less than a third of miRNAs to be highly expressed across mosquito tissues. Further, given the high number of lowly expressed miRNAs in the previtellogenic adult female mosquito, stimuli required to induce expression for the majority of miRNAs awaits discovery. Several insect studies demonstrate a wide range of stimuli can induce expression of lowly expressed miRNAs. Hormonal signals serve as developmental stimuli in both the fly and mosquito (Sempere *et al.* 2003; Liu *et al.* 2014b; Hu *et al.* 2015; Gu *et al.* 2013; Li *et al.* 2009). Environmental stimuli like insect swarm aggregation in the migratory locust, *Locusta migratoria*, induces expression of a miRNA required for synchronous egg hatching (He *et al.* 2016). Extreme cold temperature induces specific miRNA expression required for freeze tolerance in the gall fly, *Eurosta solidaginis* (Courteau *et al.* 2012). Lastly, lipopolysaccharide injection and infection with ZIKA virus serve as stimuli to induce expression of particular miRNAs in the tick, *Rhipicephalus haemaphysaloides*, and mosquito, *Ae. aegypti*, respectively (Wang *et al.* 2015; Saldaña *et al.* 2017). Thus, future work on these lowly expressed miRNAs will identify stimuli needed to induce their expression as well as decipher their importance in disease transmission by *An. gambiae*.

As human malaria continues to be one of the most important vector-borne diseases, there is always a need to learn basic mosquito physiology. Previous studies demonstrate the importance of miRNAs in mosquito reproduction (Lucas *et al.* 2015b; Ling *et al.* 2017; Zhao *et al.* 2017; Fu *et al.* 2017; Bryant *et al.* 2010; Liu *et al.* 2014a; Zhang *et al.* 2016; Lucas *et al.* 2015a) and immunity (Dennison *et al.* 2015; Winter *et al.* 2007). However, we lack basic biology behind miRNA expression in various mosquito tissues. To this end, this study yielded an updated and consolidated miRNA catalog, a genome architecture map highlighting intragenic and intergenic miRNAs, and small RNA transcriptomes for mosquito tissues critical for reproduction and immunity. As a whole, this data will provide a stronger foundation for future work on miRNAs and potentially other small RNAs in the malaria vector, *An. gambiae*.

## References

[bib1] Akbari O. S. Antoshechkin I. Amrhein H. Williams B. Diloreto R., 2013 The developmental transcriptome of the mosquito Aedes aegypti, an invasive species and major arbovirus vector. G3 (Bethesda) 3: 1493–1509. 10.1534/g3.113.006742 23833213 PMC3755910

[bib2] Allam M. Spillings B. L. Abdalla H. Mapiye D. Koekemoer L. L., 2016 Identification and characterization of microRNAs expressed in the African malaria vector Anopheles funestus life stages using high throughput sequencing. Malar. J. 15: 542 10.1186/s12936-016-1591-0 27825380 PMC5101901

[bib3] An C. Budd A. Kanost M. R. Michel K., 2011 Characterization of a regulatory unit that controls melanization and affects longevity of mosquitoes. Cell. Mol. Life Sci. 68: 1929–1939. 10.1007/s00018-010-0543-z 20953892 PMC3070200

[bib4] Attardo G. M. Hansen I. A. Raikhel A. S., 2005 Nutritional regulation of vitellogenesis in mosquitoes: implications for anautogeny. Insect Biochem. Mol. Biol. 35: 661–675. 10.1016/j.ibmb.2005.02.013 15894184

[bib5] Axtell M. J., 2013 ShortStack: comprehensive annotation and quantification of small RNA genes. RNA 19: 740–751. 10.1261/rna.035279.112 23610128 PMC3683909

[bib6] Berezikov E., 2011 Evolution of microRNA diversity and regulation in animals. Nat. Rev. Genet. 12: 846–860. 10.1038/nrg3079 22094948

[bib7] Berezikov E. Robine N. Samsonova A. Westholm J. O. Naqvi A., 2011 Deep annotation of Drosophila melanogaster microRNAs yields insights into their processing, modification, and emergence. Genome Res. 21: 203–215. 10.1101/gr.116657.110 21177969 PMC3032924

[bib8] Biryukova I. Ye T. Levashina E., 2014 Transcriptome-wide analysis of microRNA expression in the malaria mosquito Anopheles gambiae. BMC Genomics 15: 557 10.1186/1471-2164-15-557 24997592 PMC4112208

[bib9] Bryant B. Macdonald W. Raikhel A. S., 2010 microRNA miR-275 is indispensable for blood digestion and egg development in the mosquito Aedes aegypti. Proc. Natl. Acad. Sci. USA 107: 22391–22398. 10.1073/pnas.1016230107 21115818 PMC3012520

[bib10] Carthew R. W. Agbu P. Giri R., 2017 MicroRNA function in Drosophila melanogaster. Semin. Cell Dev. Biol. 65: 29–37. 10.1016/j.semcdb.2016.03.015 27000418 PMC5660628

[bib11] Castellano L. Rizzi E. Krell J. Di Cristina M. Galizi R., 2015 The germline of the malaria mosquito produces abundant miRNAs, endo-siRNAs, piRNAs and 29-nt small RNAs. BMC Genomics 16: 100 10.1186/s12864-015-1257-2 25766668 PMC4345017

[bib12] Catteruccia F. Crisanti A. Wimmer E. A., 2009 Transgenic technologies to induce sterility. Malar. J. 8: S7 10.1186/1475-2875-8-S2-S7 PMC2777329 19917077

[bib13] Chen C. Z. Li L. Lodish H. F. Bartel D. P., 2004 MicroRNAs modulate hematopoietic lineage differentiation. Science 303: 83–86. 10.1126/science.1091903 14657504

[bib14] Christodoulou F. Raible F. Tomer R. Simakov O. Trachana K., 2010 Ancient animal microRNAs and the evolution of tissue identity. Nature 463: 1084–1088. 10.1038/nature08744 20118916 PMC2981144

[bib15] Clements A. N., 2000 *Development*, *Nutrition and Reproduction*, (The Biology of Mosquitoes), Vol. 1 CABI Publishing, Wallingford, Oxon.

[bib16] Courteau L. A. Storey K. B. Morin P. Jr, 2012 Differential expression of microRNA species in a freeze tolerant insect, Eurosta solidaginis. Cryobiology 65: 210–214. 10.1016/j.cryobiol.2012.06.005 22765989

[bib17] Czech B. Hannon G. J., 2011 Small RNA sorting: matchmaking for Argonautes. Nat. Rev. Genet. 12: 19–31. 10.1038/nrg2916 21116305 PMC3703915

[bib18] Dennison N. J. BenMarzouk-Hidalgo O. J. Dimopoulos G., 2015 MicroRNA-regulation of Anopheles gambiae immunity to Plasmodium falciparum infection and midgut microbiota. Dev. Comp. Immunol. 49: 170–178. 10.1016/j.dci.2014.10.016 25445902 PMC4447300

[bib19] Friedländer M. R. Mackowiak S. D. Li N. Chen W. Rajewsky N., 2012 miRDeep2 accurately identifies known and hundreds of novel microRNA genes in seven animal clades. Nucleic Acids Res. 40: 37–52. 10.1093/nar/gkr688 21911355 PMC3245920

[bib20] Fu X. Dimopoulos G. Zhu J., 2017 Association of microRNAs with Argonaute proteins in the malaria mosquito Anopheles gambiae after blood ingestion. Sci. Rep. 7: 6493 10.1038/s41598-017-07013-1 28747726 PMC5529372

[bib21] Giraldo-Calderón G. I. Emrich S. J. MacCallum R. M. Maslen G. Dialynas E., 2015 VectorBase: an updated bioinformatics resource for invertebrate vectors and other organisms related with human diseases. Nucleic Acids Res. 43: D707–D713. 10.1093/nar/gku1117 25510499 PMC4383932

[bib22] Gruber, A. R., R. Lorenz, S. H. Bernhart, R. Neubock, and I. L. Hofacker, 2008 The Vienna RNA websuite. Nucleic Acids Res 36 (Web Server issue):W70–74. 10.1093/nar/gkn188 PMC2447809 18424795

[bib23] Gu J. Hu W. Wu J. Zheng P. Chen M., 2013 miRNA genes of an invasive vector mosquito, Aedes albopictus. PLoS One 8: e67638 10.1371/journal.pone.0067638 23840875 PMC3698096

[bib24] He J. Chen Q. Wei Y. Jiang F. Yang M., 2016 MicroRNA-276 promotes egg-hatching synchrony by up-regulating brm in locusts. Proc. Natl. Acad. Sci. USA 113: 584–589. 10.1073/pnas.1521098113 26729868 PMC4725505

[bib25] Hu W. Criscione F. Liang S. Tu Z., 2015 MicroRNAs of two medically important mosquito species: Aedes aegypti and Anopheles stephensi. Insect Mol. Biol. 24: 240–252. 10.1111/imb.12152 25420875 PMC4361387

[bib26] Jain S. Rana V. Shrinet J. Sharma A. Tridibes A., 2014 Blood feeding and Plasmodium infection alters the miRNome of Anopheles stephensi. PLoS One 9: e98402 10.1371/journal.pone.0098402 24866389 PMC4035286

[bib27] Jain S. Rana V. Tridibes A. Sunil S. Bhatnagar R. K., 2015 Dynamic expression of miRNAs across immature and adult stages of the malaria mosquito Anopheles stephensi. Parasit. Vectors 8: 179 10.1186/s13071-015-0772-y 25888742 PMC4418096

[bib28] Johnson N. R. Yeoh J. M. Coruh C. Axtell M. J., 2016 Improved Placement of Multi-mapping Small RNAs. G3 (Bethesda) 6: 2103–2111. 10.1534/g3.116.030452 27175019 PMC4938663

[bib29] Klattenhoff C. Bratu D. P. McGinnis-Schultz N. Koppetsch B. S. Cook H. A., 2007 Drosophila rasiRNA pathway mutations disrupt embryonic axis specification through activation of an ATR/Chk2 DNA damage response. Dev. Cell 12: 45–55. 10.1016/j.devcel.2006.12.001 17199040

[bib30] Kozomara A. Griffiths-Jones S., 2014 miRBase: annotating high confidence microRNAs using deep sequencing data. Nucleic Acids Res. 42: D68–D73. 10.1093/nar/gkt1181 24275495 PMC3965103

[bib31] Kugler J. M. Verma P. Chen Y. W. Weng R. Cohen S. M., 2013 miR-989 is required for border cell migration in the Drosophila ovary. PLoS One 8: e67075. Erratum .org/10.1371/annotation/709181e7-dfc9-40e9-bd0b-bd1c5541c2b1. 10.1371/journal.pone.0067075 23843983 PMC3700948

[bib32] Lampe L. Levashina E. A., 2018 MicroRNA Tissue Atlas of the Malaria Mosquito Anopheles gambiae. G3 (Bethesda) 8: 185–193. 10.1534/g3.117.300170 29146584 PMC5765347

[bib33] Lee R. C. Feinbaum R. L. Ambros V., 1993 The C. elegans heterochronic gene lin-4 encodes small RNAs with antisense complementarity to lin-14. Cell 75: 843–854. 10.1016/0092-8674(93)90529-Y 8252621

[bib34] Li S. Mead E. A. Liang S. Tu Z., 2009 Direct sequencing and expression analysis of a large number of miRNAs in Aedes aegypti and a multi-species survey of novel mosquito miRNAs. BMC Genomics 10: 581 10.1186/1471-2164-10-581 19961592 PMC2797818

[bib35] Ling L. Kokoza V. A. Zhang C. Aksoy E. Raikhel A. S., 2017 MicroRNA-277 targets insulin-like peptides 7 and 8 to control lipid metabolism and reproduction in Aedes aegypti mosquitoes. Proc. Natl. Acad. Sci. USA 114: E8017–E8024. 10.1073/pnas.1710970114 28874536 PMC5617303

[bib36] Liu N. Okamura K. Tyler D. M. Phillips M. D. Chung W. J., 2008 The evolution and functional diversification of animal microRNA genes. Cell Res. 18: 985–996. 10.1038/cr.2008.278 18711447 PMC2712117

[bib37] Liu S. Lucas K. J. Roy S. Ha J. Raikhel A. S., 2014 a Mosquito-specific microRNA-1174 targets serine hydroxymethyltransferase to control key functions in the gut. Proc. Natl. Acad. Sci. USA 111: 14460–14465. 10.1073/pnas.1416278111 25246546 PMC4209991

[bib38] Liu W. Hao Z. Huang L. Chen L. Wei Q., 2017 Comparative expression profile of microRNAs in Anopheles anthropophagus midgut after blood-feeding and Plasmodium infection. Parasit. Vectors 10: 86 10.1186/s13071-017-2027-6 28209211 PMC5314681

[bib39] Liu W. Huang H. Xing C. Li C. Tan F., 2014 b Identification and characterization of the expression profile of microRNAs in Anopheles anthropophagus. Parasit. Vectors 7: 159 10.1186/1756-3305-7-159 24690438 PMC4022070

[bib40] Lucas K. J. Roy S. Ha J. Gervaise A. L. Kokoza V. A., 2015 a MicroRNA-8 targets the Wingless signaling pathway in the female mosquito fat body to regulate reproductive processes. Proc. Natl. Acad. Sci. USA 112: 1440–1445. 10.1073/pnas.1424408112 25605933 PMC4321257

[bib41] Lucas K. J. Zhao B. Roy S. Gervaise A. L. Raikhel A. S., 2015 b Mosquito-specific microRNA-1890 targets the juvenile hormone-regulated serine protease JHA15 in the female mosquito gut. RNA Biol. 12: 1383–1390. 10.1080/15476286.2015.1101525 26488481 PMC4829293

[bib42] Marco A. Ninova M. Ronshaugen M. Griffiths-Jones S., 2013 Clusters of microRNAs emerge by new hairpins in existing transcripts. Nucleic Acids Res. 41: 7745–7752. 10.1093/nar/gkt534 23775791 PMC3763532

[bib43] Marinotti O. Calvo E. Nguyen Q. K. Dissanayake S. Ribeiro J. M., 2006 Genome-wide analysis of gene expression in adult Anopheles gambiae. Insect Mol. Biol. 15: 1–12. 10.1111/j.1365-2583.2006.00610.x 16469063

[bib44] Mead E. A. Tu Z., 2008 Cloning, characterization, and expression of microRNAs from the Asian malaria mosquito, Anopheles stephensi. BMC Genomics 9: 244 10.1186/1471-2164-9-244 18500992 PMC2430712

[bib45] Metsalu T. Vilo J., 2015 ClustVis: a web tool for visualizing clustering of multivariate data using Principal Component Analysis and heatmap. Nucleic Acids Res. 43: W566–W570. 10.1093/nar/gkv468 25969447 PMC4489295

[bib46] Mohammed J. Siepel A. Lai E. C., 2014 Diverse modes of evolutionary emergence and flux of conserved microRNA clusters. RNA 20: 1850–1863. 10.1261/rna.046805.114 25332374 PMC4238352

[bib47] Montell D. J. Yoon W. H. Starz-Gaiano M., 2012 Group choreography: mechanisms orchestrating the collective movement of border cells. Nat. Rev. Mol. Cell Biol. 13: 631–645. 10.1038/nrm3433 23000794 PMC4099007

[bib48] Nault R. Fader K. A. Zacharewski T., 2015 RNA-Seq *vs.* oligonucleotide array assessment of dose-dependent TCDD-elicited hepatic gene expression in mice. BMC Genomics 16: 373 10.1186/s12864-015-1527-z 25958198 PMC4456707

[bib49] Okamura K. Hagen J. W. Duan H. Tyler D. M. Lai E. C., 2007 The mirtron pathway generates microRNA-class regulatory RNAs in Drosophila. Cell 130: 89–100. 10.1016/j.cell.2007.06.028 17599402 PMC2729315

[bib50] Pane A. Wehr K. Schupbach T., 2007 zucchini and squash encode two putative nucleases required for rasiRNA production in the Drosophila germline. Dev. Cell 12: 851–862. 10.1016/j.devcel.2007.03.022 17543859 PMC1945814

[bib51] Quah S. Breuker C. J. Holland P. W., 2015 A Diversity of Conserved and Novel Ovarian MicroRNAs in the Speckled Wood (Pararge aegeria). PLoS One 10: e0142243 10.1371/journal.pone.0142243 26556800 PMC4640560

[bib52] Reinhart B. J. Slack F. J. Basson M. Pasquinelli A. E. Bettinger J. C., 2000 The 21-nucleotide let-7 RNA regulates developmental timing in Caenorhabditis elegans. Nature 403: 901–906. 10.1038/35002607 10706289

[bib53] Rodriguez A. Griffiths-Jones S. Ashurst J. L. Bradley A., 2004 Identification of mammalian microRNA host genes and transcription units. Genome Res. 14: 1902–1910. 10.1101/gr.2722704 15364901 PMC524413

[bib54] Saldaña M. A. Etebari K. Hart C. E. Widen S. G. Wood T. G., 2017 Zika virus alters the microRNA expression profile and elicits an RNAi response in Aedes aegypti mosquitoes. PLoS Negl. Trop. Dis. 11: e0005760 10.1371/journal.pntd.0005760 28715413 PMC5531668

[bib55] Schmittgen T. D. Livak K. J., 2008 Analyzing real-time PCR data by the comparative C(T) method. Nat. Protoc. 3: 1101–1108. 10.1038/nprot.2008.73 18546601

[bib56] Sempere L. F. Sokol N. S. Dubrovsky E. B. Berger E. M. Ambros V., 2003 Temporal regulation of microRNA expression in Drosophila melanogaster mediated by hormonal signals and broad-Complex gene activity. Dev. Biol. 259: 9–18. 10.1016/S0012-1606(03)00208-2 12812784

[bib57] Sherstyuk V. V. Medvedev S. P. Elisaphenko E. A. Vaskova E. A. Ri M. T., 2017 Genome-wide profiling and differential expression of microRNA in rat pluripotent stem cells. Sci. Rep. 7: 2787 10.1038/s41598-017-02632-0 28584262 PMC5459850

[bib58] Skalsky R. L. Vanlandingham D. L. Scholle F. Higgs S. Cullen B. R., 2010 Identification of microRNAs expressed in two mosquito vectors, Aedes albopictus and Culex quinquefasciatus. BMC Genomics 11: 119 10.1186/1471-2164-11-119 20167119 PMC2834634

[bib59] Slotkin R. K., 2018 The case for not masking away repetitive DNA. Mob. DNA 9: 15 10.1186/s13100-018-0120-9 29743957 PMC5930866

[bib60] Sokol N. S. Ambros V., 2005 Mesodermally expressed Drosophila microRNA-1 is regulated by Twist and is required in muscles during larval growth. Genes Dev. 19: 2343–2354. 10.1101/gad.1356105 16166373 PMC1240043

[bib61] Stark A. Brennecke J. Bushati N. Russell R. B. Cohen S. M., 2005 Animal MicroRNAs confer robustness to gene expression and have a significant impact on 3′UTR evolution. Cell 123: 1133–1146. 10.1016/j.cell.2005.11.023 16337999

[bib62] Theurkauf W. E. Klattenhoff C. Bratu D. P. McGinnis-Schultz N. Koppetsch B. S., 2006 rasiRNAs, DNA damage, and embryonic axis specification. Cold Spring Harb. Symp. Quant. Biol. 71: 171–180. 10.1101/sqb.2006.71.066 17381294

[bib63] Vagin V. V. Sigova A. Li C. Seitz H. Gvozdev V., 2006 A distinct small RNA pathway silences selfish genetic elements in the germline. Science 313: 320–324. 10.1126/science.1129333 16809489

[bib64] Wang F. Gong H. Zhang H. Zhou Y. Cao J., 2015 Lipopolysaccharide-Induced Differential Expression of miRNAs in Male and Female Rhipicephalus haemaphysaloides Ticks. PLoS One 10: e0139241 10.1371/journal.pone.0139241 26430879 PMC4592253

[bib65] Wang Z. Gerstein M. Snyder M., 2009 RNA-Seq: a revolutionary tool for transcriptomics. Nat. Rev. Genet. 10: 57–63. 10.1038/nrg2484 19015660 PMC2949280

[bib66] Wen J. Mohammed J. Bortolamiol-Becet D. Tsai H. Robine N., 2014 Diversity of miRNAs, siRNAs, and piRNAs across 25 Drosophila cell lines. Genome Res. 24: 1236–1250. 10.1101/gr.161554.113 24985917 PMC4079977

[bib67] Westholm J. O. Lai E. C., 2011 Mirtrons: microRNA biogenesis via splicing. Biochimie 93: 1897–1904. 10.1016/j.biochi.2011.06.017 21712066 PMC3185189

[bib68] Wheeler B. M. Heimberg A. M. Moy V. N. Sperling E. A. Holstein T. W., 2009 The deep evolution of metazoan microRNAs. Evol. Dev. 11: 50–68. 10.1111/j.1525-142X.2008.00302.x 19196333

[bib69] Winter F. Edaye S. Huttenhofer A. Brunel C., 2007 Anopheles gambiae miRNAs as actors of defence reaction against Plasmodium invasion. Nucleic Acids Res. 35: 6953–6962. 10.1093/nar/gkm686 17933784 PMC2175301

[bib70] Zhang X. Aksoy E. Girke T. Raikhel A. S. Karginov F. V., 2017 Transcriptome-wide microRNA and target dynamics in the fat body during the gonadotrophic cycle of Aedes aegypti. Proc. Natl. Acad. Sci. USA 114: E1895–E1903. 10.1073/pnas.1701474114 28223504 PMC5347622

[bib71] Zhang Y. Zhao B. Roy S. Saha T. T. Kokoza V. A., 2016 microRNA-309 targets the Homeobox gene SIX4 and controls ovarian development in the mosquito Aedes aegypti. Proc. Natl. Acad. Sci. USA 113: E4828–E4836. 10.1073/pnas.1609792113 27489347 PMC4995966

[bib72] Zhao B. Lucas K. J. Saha T. T. Ha J. Ling L., 2017 MicroRNA-275 targets sarco/endoplasmic reticulum Ca2+ adenosine triphosphatase (SERCA) to control key functions in the mosquito gut. PLoS Genet. 13: e1006943 10.1371/journal.pgen.1006943 28787446 PMC5560755

